# Supplementary figures and images for: Bioactive potentiality of secondary metabolites from endophytic bacteria against SARS-COV-2: An in-silico approach
Source: PLoS One. 2022 Aug 4;17(8):e0269962. doi: 10.1371/journal.pone.0269962 (PMC9352062; doi:10.1371/journal.pone.0269962)

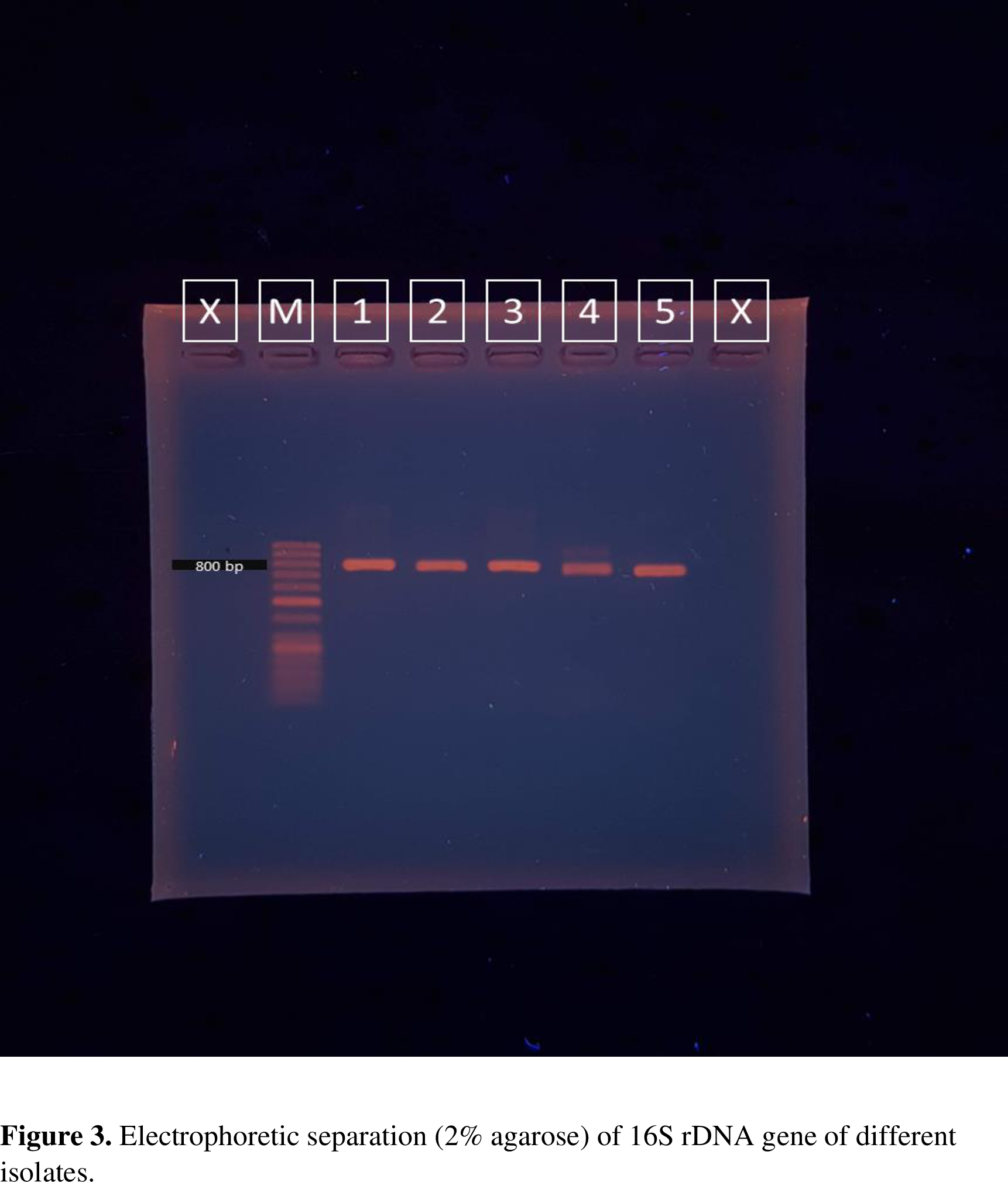

Supplement: S1 Raw images — (TIF) [file pone.0269962.s001.tif]

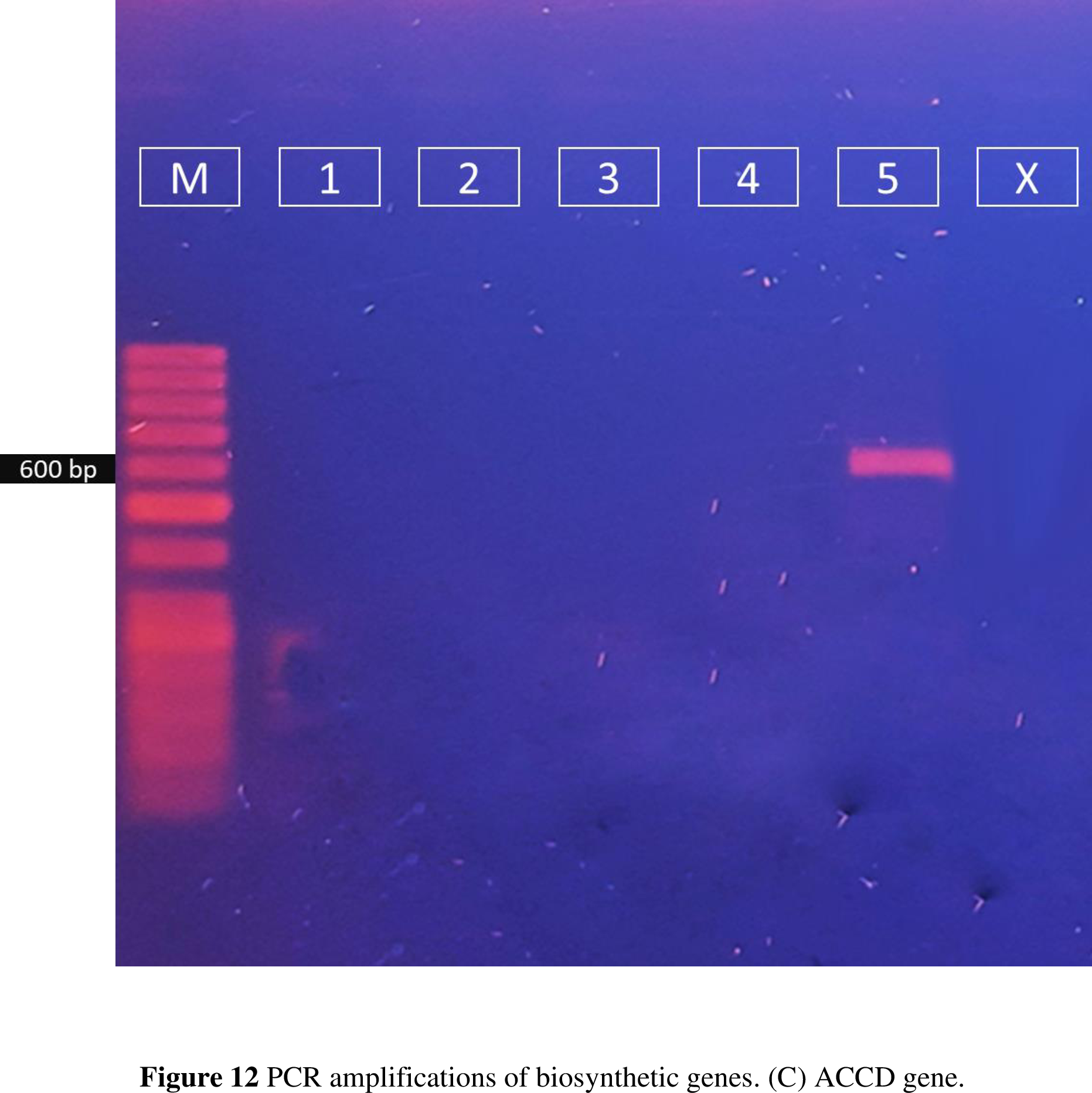

Supplement: S2 Raw images — (C) ACCD gene. (TIF) [file pone.0269962.s002.tif]

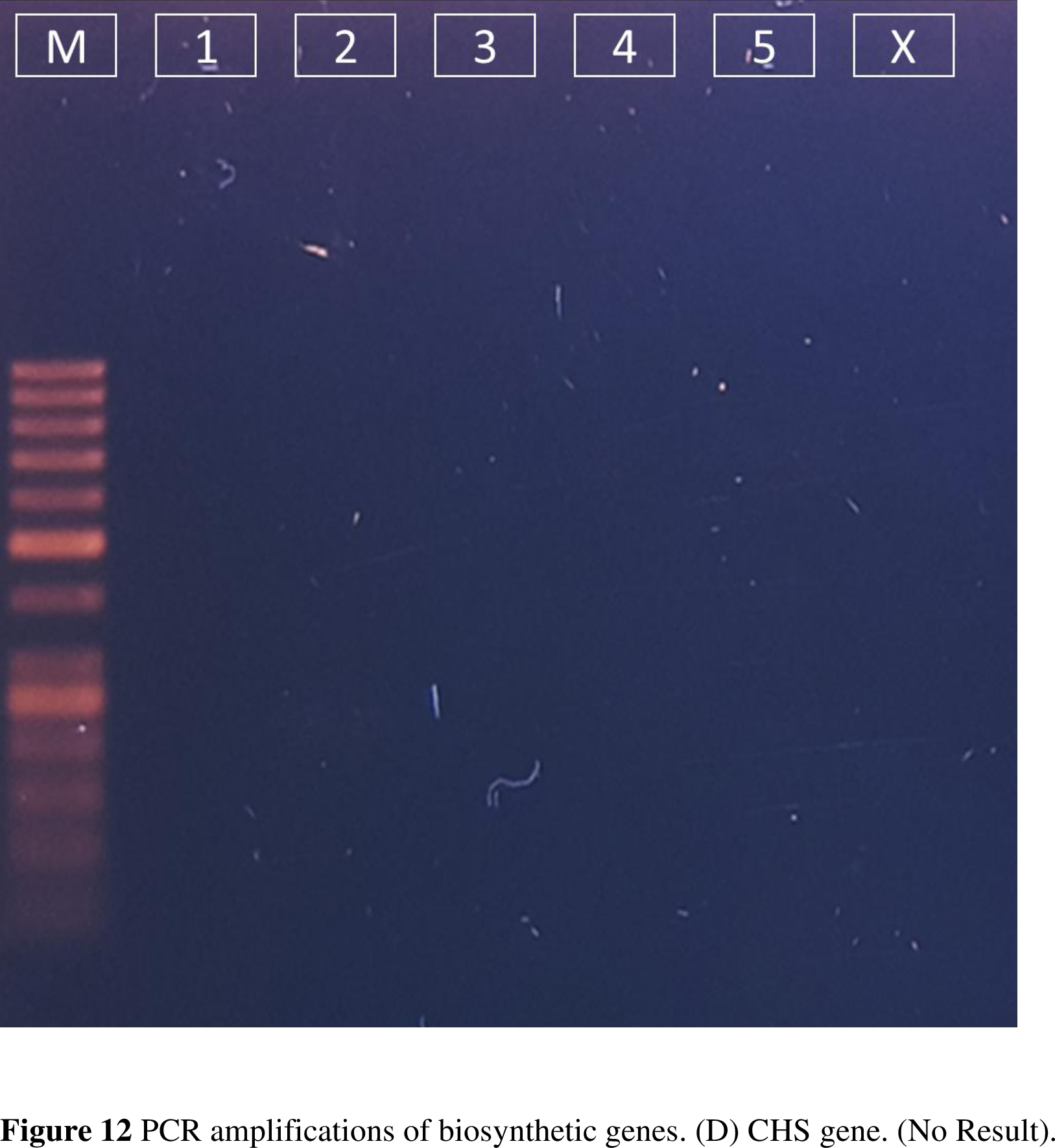

Supplement: S3 Raw images — (D) CHS gene (No Result). (TIF) [file pone.0269962.s003.tif]

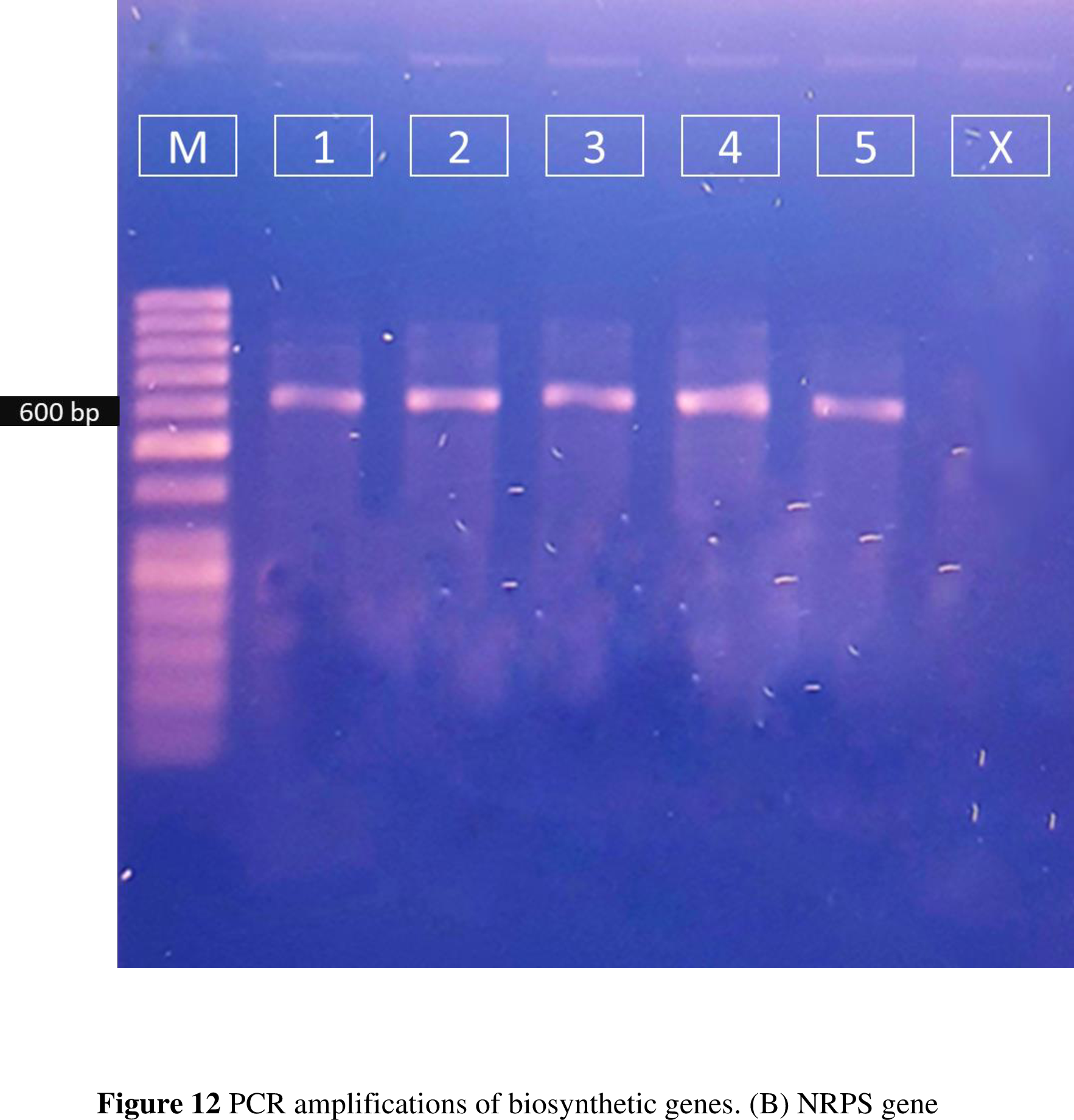

Supplement: S4 Raw images — (B) NRPS gene. (TIF) [file pone.0269962.s004.tif]

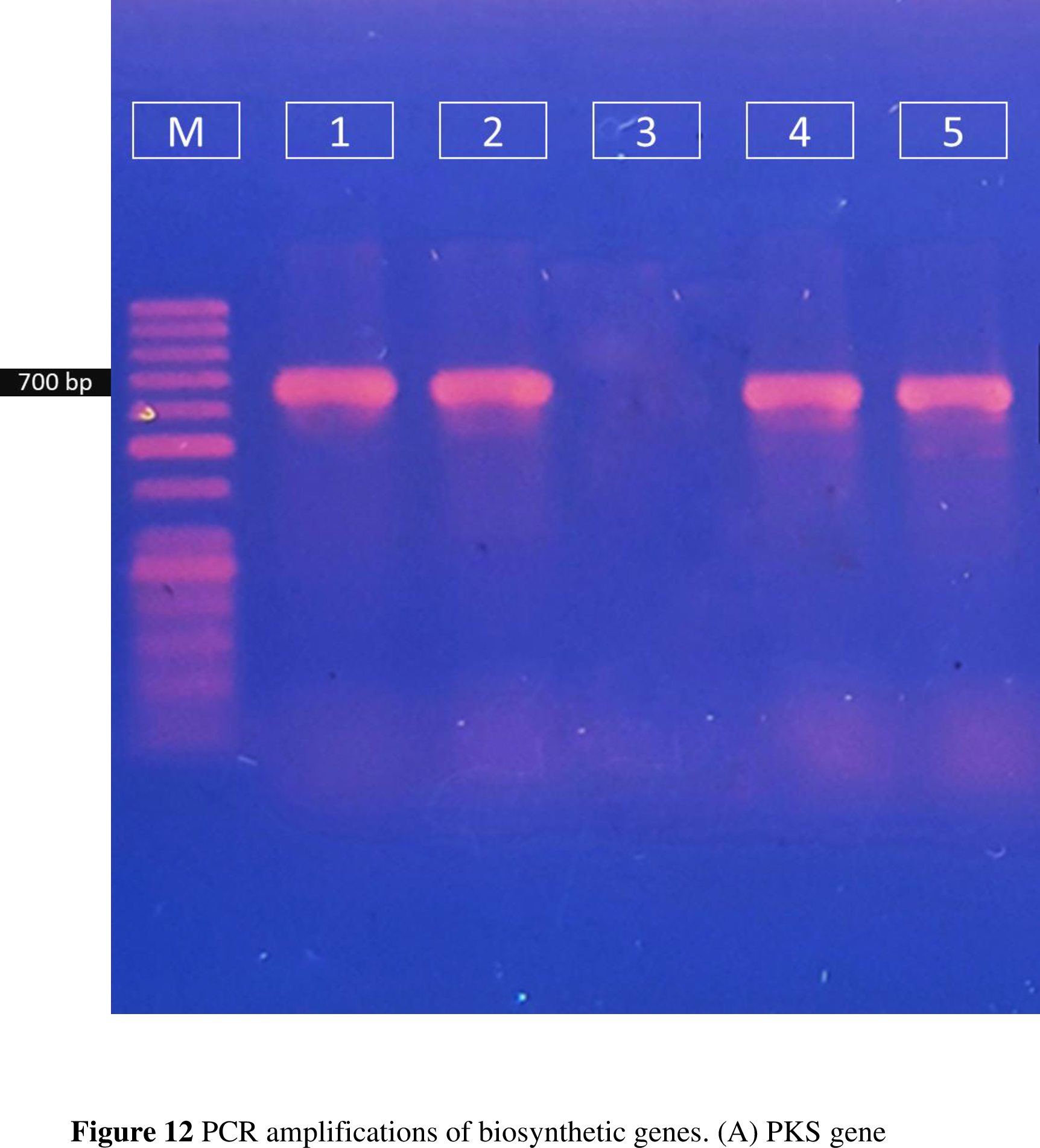

Supplement: S5 Raw images — (A) PKS gene. (TIF) [file pone.0269962.s005.tif]

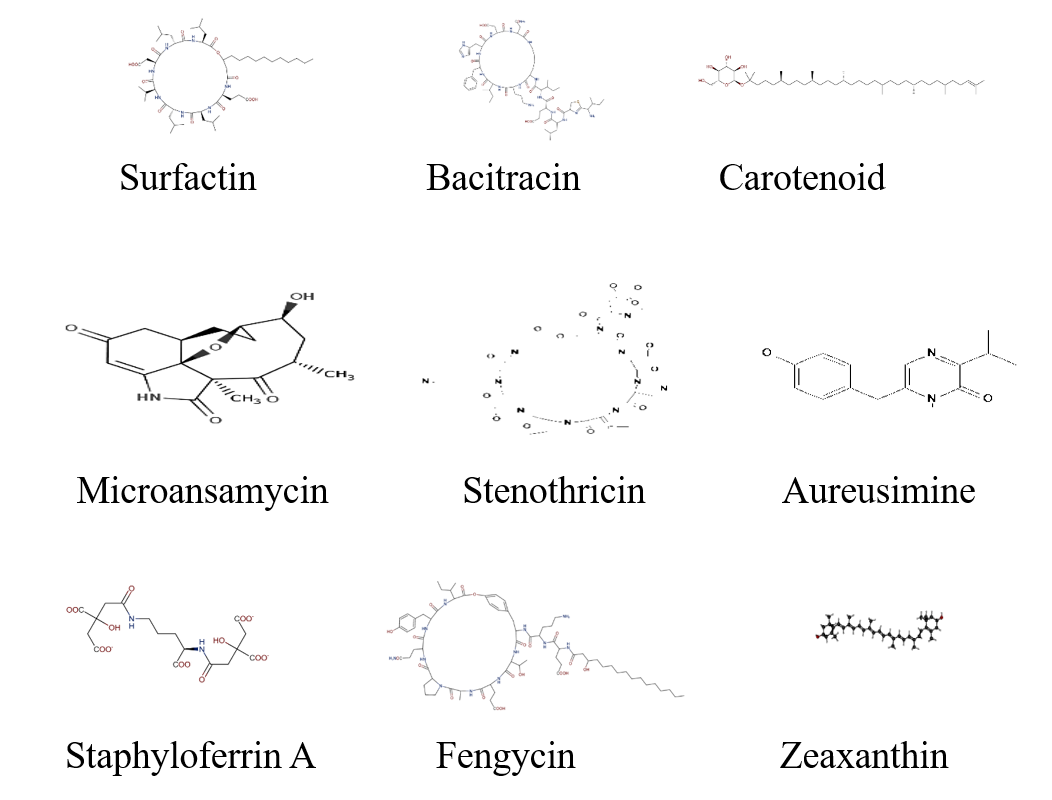


**Fig 1.** All the compounds’ structures in 3D.

Supplement: S1 Fig — (DOCX) [file pone.0269962.s006.docx]
