# Supplementary material for: Bioactive potentiality of secondary metabolites from endophytic bacteria against SARS-COV-2: An in-silico approach
Source: PLoS One. 2022 Aug 4;17(8):e0269962. doi: 10.1371/journal.pone.0269962 (PMC9352062; doi:10.1371/journal.pone.0269962)
Supplement: Attachment — Submitted filename: Plos one review.pdf [file pone.0269962.s007.pdf]

Comments on Manuscript Number PONE-D-22-01192

In silico potentiality of secondary metabolites from endophytic bacteria against SARS-COV-2

Major Concerns-

1. Although authors of manuscript are claiming that their study is related with potentiality of secondary metabolites from endophytic bacteria against SARS-COV-2, but this claim seems far from reality after observing following facts-

(a) Authors have been started collecting plant samples since 15/07/2019 and 9 sample out of 16 were collected before Dec. 2019 which they referred as the date of first case of covid-19. So, it is quite unreliable that the study was started with the aim to produce potential candidates against SARS-COV-2.

(b) Surprisingly, after doing rigorous experimentation on 16 plants and 48 plant parts they got positive results only for five plants which they started since Feb. 2020 and so on. It is giving an indication that 9 plants which have been reported in manuscript before Feb. 2020 are just to enhance data in the manuscript, although this indication can be completely false but impression of the results is like this only.

(c) Authors have been claimed activity against SARS-COV-2, the ligand they used for docking is based on their prediction of secondary metabolites from different servers (antiSMASH etc…). But the major concern in this study is that, they have been characterized the bacterial species by experimentation but after that everything is just based on prediction.

(d) On first stage they predicted the secondary metabolites present in their characterized bacterial species. Predictions may be far from reality.

(e) But still if we assume that these predicted secondary metabolites are very near to the reality (which is generally not true), further they predicted their activity against SARS-COV-2 through molecular docking and drug likeness properties. The major concern in this docking protocol is that the research and available database in the servers is far too limited to claim promising results as most of the researches are in primitive level through out the world. If one wants to predict for the disease which is existed for the last many years then due to sound database available online; these database servers and software's mostly show promising results which is rarely possible in the case of SARS-COV-2. So, if authors want to claim it, they should first find out true secondary metabolites rather than predicted and may enhance the work further. Otherwise, they may change claim of their work via rewriting the manuscript accordingly.

(f) Lines 138-143- "It was initially detected in December 2019 in Wuhan, China [31], and on 11 March 2020, the outbreak was declared by the World Health Organization (WHO) as a global pandemic that has caused a total death of 4,644,740 worldwide as of September 15, 2021, with nearly 4 million new cases reported globally in the **past week (6-12 September)** according to the World Health Organization. Unfortunately, there is no particular medication or therapy for COVID-19 at this time." We received your manuscript in Jan. 2022 and these lines are extremely confusing and again indicating that the study may not aimed towards SARS-COV-2.

2. Authors are informing that "Five biochemically identified bacterial isolates were then sent for sequencing (Macrogen, South Korea)." This is the most important part of

their study which they outsourced from South Korea. This is a major concern of the study.

3. As per table 6 they provisionally identified bacterial strains but actual findings are far from their assumptions, so how they will counter this gap in the study?
4. Introduction and discussion part is far too long concerning research article and must be rewritten thoroughly and concisely.
5. Authors are claiming “Determination of antioxidant compounds from crude extracts”, but in the discussion part they did not mention the importance and future prospects of this study.
6. Figure 15 is not self-explanatory, they referred it in Drug likeness and surely it is but it is not understandable without any explanation. Authors may omit this figure or should explain it thoroughly.

Minor concerns-

1. Lines 68-71 “Natural compounds from endophytes exhibit activity as antimicrobials, antifungals, anticarcinogens, immunosuppressants, or antioxidants; thus, secondary metabolites produced by endophytes can be implemented as therapeutics in the pharmaceutical and agricultural industries [3,4]”
   Separate references are needed to claim every other activity (primary reference must be used). The same comment applied for lines 100-102, 119-120, 126-127, 541-543.
2. In Table-2 Location (around CU campus) is given as separate entry which I feel is not much important for international research community, it may be useful for local people available there in author’s country.
3. At many places English grammar is not correct and should be checked again thoroughly.
4. Line 395- heading must be in uniform font and style.
5. Lines 537-539 “Therefore, one of the major objectives of this study is to isolate and characterize endophytic bacteria from our local medicinal plants to study their secondary metabolites, which have a wide range of applications in **our health sector**”, rather than only concentrate on their health sector, Authors should show their concern globally as they are claiming study related with a global pandemic.
