# Supplementary material for: Bioactive potentiality of secondary metabolites from endophytic bacteria against SARS-COV-2: An in-silico approach
Source: PLoS One. 2022 Aug 4;17(8):e0269962. doi: 10.1371/journal.pone.0269962 (PMC9352062; doi:10.1371/journal.pone.0269962)
Supplement: Attachment — Submitted filename: Response to Reviewer #2 .docx [file pone.0269962.s008.docx]

**Response to reviewer #2**

We would like to thank reviewer#2 for raising the following issues.

Please find our response below on manuscript number PONE-D-22-01192: ‘*In silico* potentiality of secondary metabolites from endophytic bacteria against SARS-COV-2’

The authors presented interesting research area. The *in silico* techniques the authors employed are to be appreciated. However, the manuscript needs more modification to meet PLOS ONE’s requirements.

1. It lacks consistency of ideas.

Response: Thanks for your suggestions. We have improved and modified our manuscript as per the reviewer’s instructions.

2. Scientific ideas are appeared without citation or the authors used a reference for three to six lines of scientific ideas. It should be re written.

Response: We would like to thank the reviewer for this suggestion. We have rechecked the manuscript and corrected the error accordingly.

3. The ideas between line numbers 89 and 90, that of 91 has no relationship. It is not clear how the authors write the first two lines.

Response:  We thank the honorable reviewer for valuable feedback. We have rechecked the manuscript and deleted these two lines accordingly.

4. Surprisingly the authors used wrong references. Example the reference used to cite SwissADME in the method section of the manuscript is totally wrong.

Response:  We would like to thank the reviewer for this suggestion. We have modified our manuscript as per the reviewer’s instruction. Sorry for the inconvenience.

5. The method section of the presented manuscript suffers from appropriate method writing and citations.

Response:  We have rechecked the manuscript and corrected the error accordingly.

6. The authors reported that Microansamycin (-7.7 kcal mol-1) and Aureusimine (-6.0 kcal.mol-1) are good against the studied target. However, they are not found to show interaction with Cys amino acid residue. How could they suggest the compounds are good against the studied target without interaction with Cys amino acid?

Response:  Although Cysteine residue is not found in our targeted docking sites, other residues like asparagine, lysine, threonine, serine, etc. all are having multiple H-bond with the metabolites. That’s why it gives a strong interaction.

7. Abstract and conclusions are not presented in an appropriate fashion with data support.

Response:  We would like to thank the reviewer for this suggestion. We have rechecked the manuscript and modified it accordingly.

I suggest this paper has to be resubmitted.

Response:  Thanks for your suggestion. After all modifications, we have resubmitted our manuscript again.

……
